# Protein design and RNA design: Perspectives

**DOI:** 10.1002/qub2.70029

**Published:** 2025-12-22

**Authors:** Xi Chen, Xu Dai, Peilong Lu

**Affiliations:** ^1^ Research Center for Industries of the Future, School of Life Sciences Westlake University Hangzhou Zhejiang China; ^2^ Westlake Laboratory of Life Sciences and Biomedicine Hangzhou Zhejiang China; ^3^ Institute of Biology Westlake Institute for Advanced Study Hangzhou Zhejiang China

**Keywords:** deep learning, generative models, protein design, RNA design, synthetic biology

## Abstract

Advances in deep learning and generative modeling have transformed the landscape of protein and RNA design, enabling rapid and precise creation of novel biomolecules with tailored structures and functions. In protein design, generative deep learning frameworks now support backbone generation, sequence optimization, and joint sequence–structure co‐design with unprecedented accuracy. These approaches have facilitated broad applications ranging from cyclic peptide and non‐natural fold engineering to functional tool development, including small‐molecule sensing, catalytic center scaffolding, allosteric switching, intracellular logic circuits, and the targeting of intrinsically disordered proteins. Emerging therapeutic applications—such as immune cell engineering, G protein‐coupled receptor‐targeted miniproteins, receptor‐degrading binders, and thermostable antitoxins—demonstrate the translational potential of computational design. Parallel progress in RNA design, driven by enhanced 3D structure prediction models and generative algorithms, is expanding capabilities in aptamer engineering and RNA–protein complexes, despite ongoing challenges in model generalization and experimental validation. Together, these developments highlight a new era of AI‐driven molecular engineering, in which unified protein–RNA modeling, large‐scale sampling, and automated experimental pipelines will accelerate the creation of programmable biological systems and next‐generation therapeutics.

## 1 INTRODUCTION

Proteins and RNAs are the two central active macromolecules in living organisms, orchestrating essential cellular processes such as enzymatic catalysis, regulation of gene expression, and molecular interactions through their precise three‐dimensional structures and dynamic interplay [1]. Traditionally, protein and RNA modeling have largely relied on classical computational approaches, such as homology modeling, molecular docking, molecular dynamics simulations, and ab initio structure prediction and design. These approaches typically employ refined physical models and empirical rules, combined with optimized sampling strategies. However, with the rapid advancement of artificial intelligence (AI), particularly deep learning, data‐driven approaches are increasingly emerging as transformative forces in molecular design, structure prediction, and simulation. This perspective provides a concise overview of key methodologies, representative technological advancements, and prominent application scenarios in the fields of protein and RNA design from very recent years. It is asserted that particular emphasis should be placed on the manner in which AI is propelling de novo molecular design into a new era of rapid development. Finally, the emerging potential of these AI‐driven approaches in the domains of drug discovery, the development of molecular tools, and the engineering of synthetic biological systems are discussed.

## 2 PROTEIN DESIGN: METHODS AND TECHNOLOGIES

De novo protein design seeks to create entirely novel protein molecules with desired structures and functions, independent of natural templates. Progress in this field was driven by a combination of energy function optimization, structural sampling algorithms, and increasingly, AI models. The overall workflow can be broadly categorized into three core tasks: backbone generation, sequence design, and joint backbone–sequence co‐design [2]. Each task presents distinct methodological requirements, and recent years have witnessed a surge in innovative models tailored to these challenges, offering more efficient and accurate solutions for protein design.

### 2.1 Backbone generation

In the field of de novo protein design, the generation of backbone structures represents a foundational step that determines both the functionality and stability of the resulting molecule. The backbone‒comprising the main‐chain atoms (N–Cα–C)—defines the three‐dimensional scaffold of the protein, shaping its global fold and the spatial organization of potential functional sites [2]. Unlike template‐based approaches that modify existing structures, de novo backbone generation breaks free from the constraints of natural sequences and folds, thereby exploring a significantly greater design space. However, this process presents formidable challenges, including the efficient sampling of high‐dimensional conformational space, ensuring geometric and physicochemical plausibility, and maintaining the sequence designability of the resulting scaffolds.

In response to these challenges, researchers have developed a range of strategies for generating backbones. In the early stages of this field, the approaches primarily relied on fragment assembly and parametric design. Recent advancements have seen a shift towards probabilistic generative models that learn the statistical distributions of backbone geometries from natural protein structures, thereby enabling the creation of novel yet physically plausible folds. These include SCUBA (for side chain‐unknown backbone arrangement), which constructs backbones by using neural network‐form energy terms learned from natural proteins [3]. A pivotal methodological advance arose with the emergence of the diffusion model–based algorithms for protein design, notably RFdiffusion [4] and Chroma [5], which employed complementary methodologies. RFdiffusion, a denoising diffusion framework, has been demonstrated to be effective in generating functional protein backbones that satisfy complex design constraints such as symmetry, motif scaffolding, and multimeric functional motif assembly [4]. Meanwhile, Chroma facilitates the creation of diverse topologies through fragment‐free diffusion modeling [5]. Building on the foundation of RFdiffusion, RFdiffusionAA was subsequently developed as an extension that specializes in modeling assemblies involving proteins, small‐molecule ligands, nucleic acids, metals, and covalent modifications, thereby expanding the applicability of diffusion‐based approaches [6]. SCUBA‐D, a derivative of SCUBA, incorporates diffusion modeling to achieve a balance between physical plausibility and structural diversity [7].

### 2.2 Sequence design

In the context of de novo protein design, the amino acid sequence is the fundamental determinant of the foldability, stability, and functionality of the molecule, with the spatial framework provided by the backbone. The core challenge of sequence design is to infer an optimal sequence that will not only fold reliably into the given backbone structure but also exhibit the desired biophysical and functional properties. This is a high‐dimensional, structure‐constrained combinatorial optimization problem that must balance multiple factors such as thermodynamic stability, solubility, and folding [8].

Traditional tools such as RosettaDesign rely on hybrid energy functions that integrate physics terms (e.g., van der Waals interactions, electrostatics) with empirically derived and statistical potentials (e.g., backbone torsion preferences and reference energies). Despite their potency, these approaches are encumbered by limitations in terms of efficiency and the success rate of designs, particularly when navigating the vast sequence space. In contrast, recent advances leverage deep learning models trained on structural and sequence databases, significantly improving design success rates. ProteinMPNN, a graph neural network based method, rapidly generates foldable sequences by capturing the local geometric context of the backbone, offering exceptional speed, high sampling efficiency, and strong design success rates [9]. CarbonDesign employs inverse information flow and multitask learning to couple backbone‐derived structural representations with protein language models, thereby enabling the generation of sequences and side‐chain geometries that are consistent with the input structures [10]. ProGen employs an alternative approach by utilizing a Transformer‐based protein language model that has been trained on billions of protein sequences. It enables de novo generation of functional proteins by conditioning on family, function, or structural annotations, with validated applications in antibody and enzyme engineering [11]. ESM‐IF1 performs structure‐conditioned sequence design by integrating backbone geometry into a language model, enabling efficient generation of functional sequences without the necessity of task‐specific training. Despite being trained on single chains, it has been demonstrated to generalize to complexes and has shown strong performance in the field of antibody design [12]. ProtGPS extends protein language modeling to predict subcellular localization and enable compartment‐targeted sequence design, demonstrating the existence of a localization code within protein sequences, despite its disorder at the three‐dimensional structural level [13]. Evo, a genomic foundation model, captures multimodal evolutionary constraints across central dogma processes, enabling functional protein‐RNA complex design with in vitro activities and supporting cross‐modal generation of complex systems [14].

### 2.3 Joint backbone‒sequence design

In conventional de novo protein design, backbone construction and sequence generation have typically been treated as two separate stages. The construction of a stable backbone is initiated first, followed by the design of the sequence based on the established scaffold. However, this decoupled strategy frequently fails to capture the intricate coupling between sequence and structure, thereby limiting the global fitness and functional compatibility of the final design. To overcome this limitation, recent advances have introduced the concept of joint backbone–sequence design, which aims to co‐optimize structural conformation and amino acid sequence within a unified modeling framework. These approaches leverage generative models or large‐scale protein language models to learn the joint conformational space of proteins, substantially enhancing the capacity to design complex functional sites, conformational polymorphism, and non‐natural topologies [15]. For example, relaxed sequence optimization is a protein design framework that employs gradient descent‐based optimization for updating a position‐specific scoring matrix, which facilitated the efficient design of high‐quality protein backbones. The pipeline discards the relaxed sequence, instead employing ProteinMPNN to feed the backbone geometry for sequence concretization, and structural validation is carried out using AlphaFold2 or ESMFold [16]. ESM3, a 98‐billion‐parameter multimodal generative protein language model, jointly models sequence, structure, and function through a unified transformer architecture using discrete token representations for each modality. This facilitates the model’s capacity to generate novel proteins in response to complex multi‐modal prompts, as evidenced by its demonstrated capability in designing functional GFP‐like proteins [17]. Protein Generator is a RoseTTAFold‐based model that performs sequence‐space diffusion in conjunction with structure prediction to generate sequence–structure pairs under structural, stability, and functional constraints, thereby achieving high structural fidelity and broad experimental validation across diverse protein design challenges [15].

## 3 APPLICATIONS OF PROTEIN DESIGN

### 3.1 Design of protein scaffolds and assemblies

One goal of de novo protein design is to overcome the limitations of naturally occurring protein folds by designing stable, programmable backbones and functional modules with expanded potential for diverse applications. Recent progress has focused on the design of cyclic peptides, non‐natural folds, and protein‐based materials, broadening the structural and functional landscape of proteins.

Cyclic peptides are promising therapeutic scaffolds due to their enhanced proteolytic stability and constrained conformational dynamics. Deep learning–enabled pipelines now allow de novo generation of diverse macrocycles with atomic‐level accuracy. For example, AfCycDesign employs AlphaFold2 with cyclic positional encodings to generate hundreds of thousands of topologically distinct scaffolds, with numerous alignments within sub‐angstrom accuracy to experimental structures, and is optimized as binders to targets such as MDM2 and Keap1 [18]. In comparison, RFpeptides uses a generative diffusion model, which is fundamentally different from structure‐prediction‐based frameworks, to design macrocyclic binders that precisely recognize targets like GABARAP and MCL1 [19]. This method has been demonstrated to expand the designable chemical space while ensuring accurate loop closure and target complementarity.

The engineering of non‐natural protein folds has the potential to further extend design possibilities. Salveson et al. developed a scalable framework incorporating β‐, γ‐, and 19 additional backbone types, generating millions of macrocyclic scaffolds with diverse topologies and stable, low‐energy conformations [20]. These chemically diverse, protease‐stable folds offer new opportunities for the discovery of therapeutic macrocycles and inhibitors. Sun et al. achieved the accurate de novo design of D‐protein binders composed of D‐amino acids that specifically bind L‐protein targets, with crystal structures confirming the designed hetero‐chiral protein–protein interface [21].

In the design of protein‐based materials, there has been rapid advancement in the field of programmable construction of nanoscale cages and symmetric architectures. Lee et al. combined AlphaFold‐based hallucination with ProteinMPNN sequence optimization to control protein–protein interfaces and symmetry constraints, designing protein nanocages with stable internal cavities and predefined topology [22]. These structures exhibited high accuracy in AlphaFold predictions and exhibited efficient expression, purification, and assembly, thus highlighting their potential as drug delivery vehicles, vaccine carriers, and bio‐nanocontainers.

### 3.2 Design of functional protein tools

As de novo design methodologies mature, researchers are becoming increasingly proficient in encoding specific molecular functions—such as small molecule recognition, enzymatic catalysis, and conformational switching—into novel protein scaffolds. This capability not only pushes the functional boundaries of natural proteins but also provides a critical foundation for synthetic biology, diagnostics, and dynamic regulatory systems.

#### 3.2.1 Small molecule probes and binding cavities

Precisely engineered small molecule–binding pockets are essential for targeted recognition, sensing, and signaling. Recent advances have demonstrated that de novo designed proteins can bind small‐molecule ligands with high affinity and specificity, opening avenues for programmable chemical recognition in biological environments.

Lu et al. employed a physics‐based approach integrating vdM‐guided binding site sampling and molecular dynamics simulations to construct Poly (ADP‐ribose) polymerase inhibitor binders with sub‐nanomolar affinities, with crystal structures accurately recapitulating designed interactions [23]. Zhu et al. developed transmembrane proteins that activate fluorescence upon ligand binding by integrating deep learning with energy‐based methods. These transmembrane proteins, engineered by designers, exhibit strong specificity and affinity in living cells, enabling precise chemical recognition within membrane environments [24]. Lee et al. integrated deep learning with physical modeling to design binding proteins with nanomolar affinities for various ligands, successfully developing functional sensors for the detection of small molecules, such as cortisol [25].

#### 3.2.2 Catalytic center design

Designing functional enzymes from scratch is a major goal in translating de novo design into practical molecular tools. Early work by Anishchenko et al. demonstrated that deep network hallucination can generate foldable backbones while embedding geometrically constrained catalytic motifs, enabling the creation of stable proteins with designed active sites [26]. Recently, Lauko et al. developed a generalizable deep learning based strategy for constructing serine hydrolase enzymes with atomically precise catalytic triads [27]. They utilized RFdiffusion [4] and PLACER [27] to generate protein scaffolds that accurately organize active site residues throughout multistep reaction cycles. Using serine hydrolases as a model, they successfully achieved the de novo creation of enzymes featuring complex Ser‐His‐Asp catalytic triads and oxyanion holes, enabling catalytic turnover and high structural fidelity. By generating backbone geometries from minimal motifs and assessing intermediate‐state compatibility, this approach circumvents traditional scaffold limitations, yielding novel folds and improved catalytic efficiencies. The collective findings of these studies demonstrate the efficiency of deep learning strategies in achieving atom‐level control over the geometry of catalytic centers. This provides a replicable framework for the development of synthetic enzymes.

#### 3.2.3 Conformational switching and allosteric control

Dynamic regulation of protein function through conformational transitions presents a significant challenge in protein design. Pillai et al. developed an oligomeric protein system featuring a bistable hinge that reversibly switches between symmetric states upon binding of a helical peptide, thereby enabling cooperative regulation and reconfigurable assembly [28]. Guo et al. developed dynamic proteins capable of switching between distinct conformations in response to ligands or distal mutations, demonstrating controllable “on/off” switching functionality [29]. These designs serve as foundational frameworks for synthetic signal transduction and conformational switches.

Shen et al. have utilized computations designed to engineer subunits that contain multiple buried histidine residues [30]. These subunits are capable of undergoing conformational transitions driven by slight pH changes, thereby achieving highly sensitive and cooperative environmental responsiveness. The pH‐responsive protein fibers that were designed self‐assemble into micrometer‐scale ordered fibers under neutral pH conditions and exhibit rapid and reversible dissociation in response to pH changes. This design not only demonstrates the potential of protein‐based nanomaterials in environmental responsiveness, but also provides new insights for the development of synthetic regulatory systems based on conformational changes.

#### 3.2.4 Programmable protein logic circuits for intracellular signal processing

Protein‐level neural networks represent a cutting‐edge direction in synthetic biology, enabling rapid and programmable decision‐making directly within living cells. Chen et al. engineered a “winner‐take‐all” classification circuit by integrating de novo–designed heterodimers with modular protease components, thereby constructing a system that performs signal classification through weighted input summation, self‐activation, and mutual inhibition of output nodes. When implemented in mammalian cells, the system enables multi‐output decision‐making and can drive functional outcomes such as apoptosis. Bypassing transcriptional mechanisms, this protein‐based computational framework provides a rapid and portable strategy for logic‐driven cellular computation, thereby advancing the programmable control of cellular behaviors [31].

#### 3.2.5 Binder design for IDPs

Intrinsically disordered proteins (IDPs), which constitute nearly 40% of the human proteome, lack stable tertiary structures and exist as highly dynamic conformational ensembles [32]. The functional interactions between these proteins are mediated by diverse binding modes, whereby disordered regions undergo structural transitions, often being induced into binding‐competent conformations, such as extended backbones or partial secondary structures upon binding to a partner [33]. IDPs have long been considered “undruggable” by conventional structure‐based strategies. To address this challenge, Wu et al. developed Logos, a two‐stage computational framework for targeting intrinsically disordered regions (IDRs) [34]. The pipeline first constructs a diverse template library by generating repeat protein scaffolds via Rosetta and assembling specialized binding pockets using RFdiffusion. Subsequently, the IDR segments are threaded through these templates and refined to enable induced‐fit binding. Wu’s Logos framework is particularly effective for IDRs that favor binding in extended conformations. Complementarily, Liu et al. utilized RFDiffusion to design binders targeting IDP sequences, which possess the capacity to adopt partial helical or β‐strand conformations upon binding [35]. Interface formation was optimized either through two‐sided partial diffusion, which enables both the target and binder to co‐sample complementary conformations, or through secondary‐structure constraints applied when targeting shorter IDRs. In these designs, disordered targets were induced into binding‐competent states characterized by partial helical or β‐strand elements, thereby broadening the design landscape for IDP‐targeting binders across diverse sequence and structural contexts.

### 3.3 Application in therapeutic development

#### 3.3.1 Application in immune therapy

Integration of protein design into CAR‐T cell therapy facilitates precise targeting and effective killing of tumor cells. Xia et al. developed a computationally designed high‐affinity protein binder—referred to as a de novo designed binder (DNDB)—which was incorporated into CAR‐T constructs to target tumor surface antigens such as epidermal growth factor receptor (EGFR) and CD276 [36]. These DNDBs exhibit high binding affinity and low immunogenicity, addressing key biochemical limitations of traditional single‐chain variable fragments (scFvs). In various glioblastoma models, CAR‐T cells engineered with these modules demonstrated enhanced cytotoxicity, persistence, and tumor clearance capacity, along with a reduced immunosuppressive phenotype. This work highlights the potential of de novo protein design in the context of reprogramming cellular immunity.

#### 3.3.2 Venom toxin antagonist design

Snake venom toxins, owing to their molecular diversity and rapid lethality, have long been in need of highly thermally stable countermeasures—particularly valuable for use in developing countries where cold chain transportation and storage systems are limited. Torres et al. proposed a de novo protein design strategy using AlphaFold2 and RFdiffusion to construct high‐affinity protein inhibitors against snake venom components [37]. Focusing on short‐ and long‐chain α‐neurotoxins and cytotoxins within the three‐finger toxin family, the team designed complementary binding proteins by targeting peripheral β‐strands of the toxins. This involved RFdiffusion‐driven backbone generation, ProteinMPNN‐based sequence optimization, and AlphaFold2 structure validation. The resulting small neutralizing proteins significantly improved survival in murine models and effectively blocked multiple injury mechanisms—including neurotoxicity and enzymatic toxicity—while exhibiting high specificity, low immunogenicity, and favorable thermal stability. This study provides compelling evidence that protein design offers a viable and transformative path for next‐generation antivenom therapeutics.

#### 3.3.3 Application in targeting cell surface membrane proteins

Targeting receptor‐mediated endocytosis has emerged as a strategy to degrade cell surface membrane proteins. Huang et al. developed a computational approach for designing synthetic binders, termed EndoTags, which have been shown to trigger rapid internalization and lysosomal degradation of surface receptors such as EGFR. These EndoTags operate via three distinct mechanisms: Firstly, orthogonal binding to constitutively cycling receptors like TfR and Sortilin without interfering with endogenous ligands; secondly, inducing conformational changes in receptors such as IGF2R by bridging critical extracellular domains; and thirdly, receptor clustering, where multivalent EndoTags aggregate receptors like asialoglycoprotein receptor to initiate endocytosis. In both cell lines and mouse tumor models, this approach significantly suppressed tumor growth. This method is broadly applicable and provides a promising strategy for the precise degradation of target proteins on cell surfaces [38].

Edman et al. engineered geometrically tunable oligomeric scaffolds displaying FGFR1c‐specific minibinders, thereby enabling precise control over receptor clustering and downstream signaling. These synthetic agonists surpass native fibroblast growth factor ligands by offering isoform specificity, sustained signaling, and tunable receptor binding geometry. Their ability to drive cell fate decisions such as arterial versus perivascular differentiation highlights the therapeutic potential of rationally designed protein assemblies in modulating surface receptor function [39].

In addition to receptor clustering and internalization, recent advances have expanded the landscape of precise receptor binding via computationally designed miniproteins. Edin et al. reported a computational de novo design strategy in combination with high‐throughput screening to generate miniprotein agonists and antagonists for G protein‐coupled receptors (GPCRs). GPCRs play central roles in physiological regulation and drug development. However, the process of designing protein‐based agonists and antagonists has historically been fraught with difficulty due to the inherent conformational dynamics of these integral membrane proteins. The integration of computational de novo design with a “receptor diversion” microscopy‐based screening method has enabled the successful generation of agonists targeting MRGPRX1 and antagonists against CXCR4, GLP1R, GIPR, GCGR, and CGRPR. This provides a new and feasible pathway for the design of GPCR‐targeted protein therapeutics [40].

Berger et al. provided evidence for the potential of orally delivered miniproteins as a treatment for autoimmune and inflammatory diseases. The study yielded high‐affinity, highly stable miniproteins targeting IL‐23R and IL‐17. These miniproteins effectively blocked cell signaling in vitro and exhibited remarkable resistance to heat, acid, and proteolysis, thus enabling the possibility of oral delivery. In a mouse model of colitis, orally administered IL‐23R miniproteins demonstrated therapeutic efficacy [41].

Collectively, these studies demonstrate the versatility and therapeutic potential of computationally designed proteins in targeting cell surface membrane proteins. By enabling precise control over receptor binding, clustering, and internalization, these strategies allow for the modulation or degradation of disease‐relevant receptors with high specificity and tunability. Moreover, the development of orally deliverable, stable miniproteins expands the avenues for non‐invasive administration, further enhancing the translational potential of these approaches.

## 4 RNA DESIGN: PROGRESS, CHALLENGES, AND FUTURE DIRECTIONS

The field of RNA design has undergone rapid advancement in recent years, largely due to the emergence of deep learning and structural modeling tools. These tools have enabled the rational generation of sequences that exhibit the desired folding and function. Moving beyond traditional methods, data‐driven models now capture sequence–structure relationships under defined secondary or tertiary constraints, thereby improving the precision and scalability of RNA design [42]. Recent developments in 3D RNA structure prediction, including RoseTTAFoldNA [43], RoseTTAFold All‐Atom [6], AlphaFold3 [44], RhoFold+ [45] and trRoseTTARNA [46], have expanded the toolkit for RNA structure modeling. RoseTTAFoldNA extends the RoseTTAFold framework to predict RNA and protein–RNA complexes directly from sequence, providing accurate models and confidence estimates that surpass traditional docking‐based approaches. RoseTTAFold All‐Atom extends structure prediction to RNA and RNA–protein complexes within a unified all‐atom framework. AlphaFold 3 employs a diffusion‐based architecture to predict structures of diverse biomolecular complexes. RhoFold+ leverages an RNA language model with transformer‐based refinement. trRoseTTARNA, in contrast, predicts geometric restraints with a transformer network and folds structures via energy minimization. Together, these models furnish complementary approaches for integrating secondary, tertiary, and evolutionary features into RNA structure prediction pipelines.

Generative models such as variational autoencoders and reinforcement learning frameworks have shown promise in producing functional RNA motifs, including aptamers and ribozymes [47, 48, 49]. These methods often leverage family‐specific structural data or experimental feedback (e.g., SELEX) to enhance design specificity and biological relevance. Key challenges persist, including the limited generalizability across RNA families and the variability in the accuracy and success rate of 3D folding [50, 51]. The potential of designed RNAs in diagnostics, therapeutics, and synthetic biology is significant. It is notable that RNA aptamers exhibit a high degree of affinity and programmability for molecular recognition and targeted delivery [47]. Additionally, RNA‐based biosensors and nanostructures have the potential to be used for dynamic sensing and smart materials [52].

In summary, although recent RNA design technologies have significantly advanced, the path from *in silico* generation to functional application remains underdeveloped. It is imperative that future research must address model generalization, predictive accuracy, and integration with experimental pipelines to realize the full potential of synthetic RNA systems.

## 5 CONCLUSION

Protein and RNA design are entering a new era of AI‐driven precision and functional sophistication. The advent of deep learning and generative models has dramatically increased the efficiency and feasibility of backbone generation, sequence optimization, and functional coding, enabling applications that span fundamental structural innovation to drug development and synthetic biology. At the same time, the design of protein–RNA complexes is emerging as a frontier, offering novel strategies for gene regulation, RNA therapeutics, and the construction of artificial biological circuits. In the future, the combination of algorithmic advances, multiscale modeling, and high‐throughput experimental validation platforms will lead to the acceleration of molecular design towards greater efficiency, intelligence, and systems‐level control. This will have transformative impacts on biomedicine and synthetic biology.

## AUTHOR CONTRIBUTIONS

**Xi Chen**: Conceptualization; investigation; writing—original draft; writing—review and editing. **Xu Dai**: Investigation; writing—original draft; writing—review and editing. **Peilong Lu**: Conceptualization; supervision; writing—review and editing.

## CONFLICT OF INTEREST STATEMENT

The authors declare no conflicts of interest.

## ETHICS STATEMENT

This article does not contain any studies with human or animal materials performed by any of the authors.

## Data Availability

Data sharing is not applicable to this article as no new data were created or analyzed in this study.
